# A Clinical Lecture upon Cutaneous Epithelioma

**Published:** 1880-08

**Authors:** I. Edmondson Atkinson

**Affiliations:** Clinical Professor of Dermatology, University of Maryland, etc., Baltimore


### Selections.

---

A CLINICAL LECTURE UPON CUTANEOUS EPITHELIOMA. By I. EDMONDSON ATKINSON, M.D., Clinical Professor of Dermatology, University of Maryland, etc., Baltimore.

*Gentlemen:* The patient I now show you is a widow, sixty-seven years of age, living in Baltimore county. She has informed me that about seven years ago a small spot, of pin-head size, came upon her left cheek. It was then partly scaly, and would, at times, discharge a scanty, thin fluid. Two years or more ago, a similar lesion made its appearance upon the bridge of her nose. There has been no other subjective symptom accompanying these, than a slight itching. At present the spot upon the right cheek is 1 cm. in diameter. It is of a pale-red color, not ulcerating, perfectly soft, and scaly. No scar can be detected upon it nor in its vicinity. It is itchy, and, indeed, cannot be distinguished from a patch of scaly eczema. The spot upon the bridge of her nose has gradually and steadily extended, and is now 2.5 cm. in diameter, and is quite circular. It is distributed nearly symmetrically, and its borders approximate, but do not involve the inner canthi. The surface of this area is rather darker than the surrounding skin, and is, for the most part, covered with thin, desquamating epidermis. Little ulcers have, from time to time, our patient states, appeared upon this surface, and after healing, are seen to have converted it almost wholly into a superficial scar-tissue. There is now, at the lower border, a small, brownish scab, which covers a superficial ulcer, whose base can be felt to be slightly indurated. Along the right inner margin of the patch, are arranged several granules, or pearl-like nodules, no larger than mustard seed. There are scattered about this margin several other thin scabs and some epidermic accumulations. The affected area, taken as a whole, is very slightly infiltrated, and occasions no pain, but some itching.

Scattered irregularly over the upper part of the face are a few small spots, consisting of thin, gray and greasy-looking epidermic scales. These can be easily removed by the finger-nail ; and the surface thereby exposed, is seen to be moist, sticky, with here and there a tiny drop of blood. Notice, in addition, that all the glands of the neighborhood are in a perfectly healthy condition.

Finally, the patient presents an appearance of general good health, gives a healthy history, and is not aware of any member of her family having had cancer.

Neither the appearance nor the history of this patient could impress one not acquainted with the disease, as at all alarming ; and yet, we have here a form of cancer—flat, superficial carcinoma, or epithelioma of the skin.

Allow me, however, without further discussion of this case, to pass to the consideration of another form of the disease, and to read the following extracts from my notes :

Mrs. K., a German widow, sixty-four years old, of slender frame, medium height and dark complexion, came for treatment to the Special Dispensary, June 10th, 1878. Four years previously, she noticed a pimple upon the left side of her nose over the nasal bone. Slowly it became an open sore ; and last fall the ulcer was destroyed with caustics. The result was successful, and the parts healed perfectly. There was no sign of re-appearance until April.

When I first saw her, there was an ulcer, situated at the margin of a bed of scar-tissue, 1.5 cm. in diameter, just below the left inner canthus, towards the median line. It was as large as a small shirt-button, and had clean, reddish edges. The part in which it was imbedded was movable about the circumference of the ulcer, but attached to the bone at its base. Along the borders, were a few medium-sized nodules. After removing a thin, dark-brown scab, the ulcer was seen to be funnel-shaped, of a reddish-brown color, moistened by a scanty, thin discharge. The edges were not everted, but were abrupt. The whole was considerably indurated. Pressure forced out a little bloody serum. The neighboring lymphatics were not involved. The family history seemed to indicate no cancerous antecedents.

Recognizing this ulcer as an epitheliomatous one, on June 17th, the patient being anæsthetized, I spooned it out with the dermal curette, as thoroughly as possible, and satisfied myself that the subjacent nasal bone was extensively implicated. I next introduced a crayon of silver nitrate, and carried this wherever the morbid tissues would yield before it. The point sank through the diseased portion of the nasal bone, as through a cheesy substance, making a free opening into the nasal cavity as large as a goose-quill. The crayon was made to burrow in every direction, until only solid, unyielding tissues were encountered. The pultaceous mass was then turned out, and the wound dressed with antiseptic cotton. Recovery was rapid and complete, and when seen by me, October 10th, the integument was continuous, and the destruction of osseous tissue was only made evident by a depression. There was no sign whatever of a renewal of the new growth.

The patient whom I now present to you is the subject of the following extract from my note-book:

Mrs. G., an Irish widow, slender and wiry, had always enjoyed excellent health. Eight or nine years ago, a little wart appeared upon her right cheek, near her nose. It gave no trouble, and remained almost unnoticed. Three years ago, it began to enlarge. It has never given pain, except when she assumed a stooping posture, when it would throb. I saw her first July 15, 1879, at the Special Dispensary. She then had a tumor situated upon her right cheek—its inner border almost reaching to the nose. The superior margin of this growth was 1.5 cm. below the lower lid. The tumor was nearly circular and evenly dome-shaped, measuring, in vertical diameter, 2.5 cm.; transversely, 2 cm. It projected nearly 5 mm. above the line of the surrounding skin, from which it sprang abruptly. It was smooth, red and shining as if varnished. Its epidermis was continuous, except at five or six points where minute excoriations were present. These were covered with small, thin, brown or concave scabs. Beneath the epidermis, some red, sinuous lines (dilated blood-vessels) could be seen. The whole growth was freely movable, and did not involve the subcutaneous tissues. The skin, however, was affected in its whole thickness. At a few points of the periphery, very small scars were visible (from previous ulceration). The whole mass was slightly indurated. There was no glandular complication.

The diagnosis was here, again, cancer, but of the papillomatous variety—papillomatous epithelioma. Excision with the knife was here plainly practicable, but was positively declined by my patient, who also refused to submit to anæsthetization. Being thus obliged to forego the prospect of an immediate and speedy operation, I determined to have recourse to caustics. For reasons that I will presently explain to you, I selected the arsenical paste of Cosme as modified by Hebra. This paste is composed as follows, viz.:

| | | | | |
|---|---|---|---|---|
| ℞. | Acid arsenios.......................... | (gr. x.) | 0 | 6 |
| | Hydrarg. sulphuret. rub............. | (ʒss) | 2 | 0 |
| | Ung. aq. ros........................... | (℥ss) | 15 | 0 |

M. S. Arsenical paste.

This paste I applied, spread upon lint, and protected by oiled silk and a compress. It was re-applied daily for four days, when the slough which had formed was dressed with a poultice. After this slough had cleared away, a granulating surface of pinkish color and of minute size was revealed. It being evident that the cancerous growth had not been completely destroyed, the paste was, in three weeks, applied as before for four days. The result of this second cauterization was most gratifying, and, as you may now observe, cicatrization is perfect. At one point, a tiny scale, not so large as a pin-head, may indicate the return of the new growth. This is very doubtful; but I have cautioned her to watch its condition narrowly.

The next case I wish to speak of presented still another—the infiltration form of carcinoma, cancer of the lip.

This patient, Patrick F., was, doubtless, known to many of you as an inmate of this hospital last summer. He was tall, slender, of feeble aspect, and 52 years old; of Irish birth. Five years ago he had a non-parasitic sycosis, which began upon his left cheek, and speedily extended to the right side of his face. He was treated for this by Dr. Michael. It is now well, but has left a smooth surface of scar-tissue in its stead.

As, however, it is not to this feature I desire to draw your attention, I pass to another point of the case. Two years ago, he first noticed a lump in his right lower lip. This gradually increased in size, and when seen by me, July 20, 1879, it had attained the dimensions of a hickory nut, involving the entire thickness of the lip, and extending far down towards the chin, including both skin and mucous membrane. It was of gristly hardness. To the eye, it appeared as if glazed or varnished, and was superficially ulcerated. The lip was everted, and a stream of saliva dribbled down to the chin. The color of this tumor was a pale pink, and its excoriated surface was coarsely granular. A thin, serous fluid bathed it. The submaxillary glands were larger than normal, but did not give an impression of specific induration. Pain was an insignificant symptom. No family history of cancer could be obtained. This patient had upon his lip the deep-seated or infiltrating form of epithelioma.

Epithelioma—epithelial cancer of the skin—comprises three forms—the two milder ones, however, tending to run into the third, more malignant one. These are—

1. The superficial or flat variety.
2. The papillomatous variety.
3. The deep-seated or infiltrating variety.

First, let us consider a few properties held by these three forms in common.

*Cutaneous epithelioma* is a disease of advanced life, rarely occurring before the fortieth year, more frequently after the fiftieth year. It is said to be more commonly met with in males. Of all parts of the surface liable to it, the face is, beyond all comparison, the seat of election. Next in frequency, the genital organs are attacked by it; but it is not proposed to treat in this lecture, of the disease as occurring in the latter parts. There seems to be an especial predisposition for epithelioma to attack those parts where there has been a congenital or acquired epidermic malformation, such as occurs in warts, moles, etc., or to start from spots where are those little accumulated patches of epidermic and sebaceous products, such as one frequently sees upon the integument of the aged, and such as now point out to you upon the patient before you. Still further, the new growth tends to attack those individuals whose skins are, in their entirety, of defective congenital organization. This plaster cast, for example, represents a papillomatous epithelioma upon the arm of a man 60 years old, who had had a simple ichthyosis since infancy. Strange to say, hereditary transmission does not seem to play the same important role in this affection as in some others.

Epithelioma of the skin usually begins as a nodule of minute size, and of waxy appearance; or it may begin, as already mentioned, in flat, greasy-looking, scaly spots of minute size. (Both of these conditions may be found in the patient I first presented to you; the scaly spots being distributed over various portions of the face; the little wax-like points imbedded in and slightly projecting from the margin of the patches upon the nose.) Or, the disease may begin in a wart or a mole, which may have been of life-long duration or of recent appearance. At all events, the beginning is usually so insignificant, that for a long time it may fail to attract attention. This is more especially the case in the flat or superficial form of the disease. At other times, as in the case of F., where the cancer is of the deep-seated infiltrated variety, the new growth reveals itself first as a small tumor imbedded in the skin.

The mildest variety of cancer of the skin—the flat or superficial form—beginning as a tiny nodule, an abrasion, a scale, a small scab, may, and often does, exist for years without attracting more than passing notice. After several years, it may have developed a superficial ulcer no larger than a half-dime, with reddish, glazed, scantily-secreting base, often covered wi h a dark, thin crust; and slightly indurated; or, it may even be so small that ulceration will be scarcely recognizable, and yet the morbid action may have produced considerable destruction of tissue, whose place will have been usurped by a scar; for, while the ulcer may continue a small one, and always superficial, it will, in its course, have invaded a large tract—healing at one point, while invading new tissue at another.

I lately had an opportunity of seeing this form of epithelioma upon the cheek of an elderly gentleman, who had had it for a great many years. During all this time, the only active lesion had been ulceration so very superficial and limited, that it was invariably covered by a little black crust, and had only attracted attention by its pertinacity. It appeared originally near the center of the cheek; but when seen by me, it was near the tragus of the ear. But it had not made this change of location without leaving its trail behind it. The tissue between the little scab and the point of origin was no longer normal tissue. It was a superficial scar, and included a space nearly as large as a silver dollar. A noteworthy circumstance was, that the little ulcer, with its scab, had not travelled continuously, but had occasionally healed entirely at one point, to re-appear at another (and this behavior is often observed in the course of epithelioma). The new growth had been slowly infiltrating the normal tissue and causing its destruction. As repair and cicatrization took place in its wake, the disease was always progressive; and yet, in all these years, the ulcer did not increase in size and activity. It is now no more formidable in appearance than at the beginning, and is still going on as it began.

When, however, as is usually the case, in the course of time, a decided ulcer is formed, it presents peculiar characters. Its surface has a dry, shining appearance as if varnished, and its color is grayish-red or brownish-red. It is also coarsely granular. The scanty sero-sanguineous discharge tends to dry into thin crusts, and there is presented a general aspect of inactivity. The form of the ulcer is irregularly circular; its edges quite clearly defined; and to the touch there is a decided feeling of induration. Scar-tissue is frequently visible in the vicinity, attesting an attempt at repair. During this period, the limits of the corium may or may not have been exceeded; frequently, the new growth will have been limited to the superficial portions of the corium; at other times, deeper tissues have been involved. In the latter event, the disease may long retain its indolent course, or it may pass rapidly into the deep-seated, infiltrating variety, and the gravity of the prognosis will be proportionately increased.

The flat, superficial form may last for years, even as many as ten, fifteen or twenty years, and remain all the time a strictly local process, never giving rise to infection of the neighboring lymphatics. Indeed, it is impossible for the new growth to become replaced by scar-tissue entirely, and the cancer to be thus permanently cured. This desirable result, unfortunately, is not frequently realized, and sooner or later, the superficial cancer becomes deep, and its ravages more rapid and alarming.

When this ulcerative process has become pronounced, but remains indolent, this variety of epithelioma, when upon the face, was formerly, and is still by many, especially in England, known as "rodent ulcer;" and its cancerous nature has often been denied. This view is, however, based upon the slight degree of malignity attached to this form of ulcer, and to its supposed histological peculiarities, insisted upon by many English pathologists, headed by no less an authority than Sir James Paget, who deny that the new growth is of an epithelial nature. This view, however, is now quite untenable, and it is certain that "rodent ulcer" is histologically allied to and identical with carcinoma. The clinical relations, indeed, of this form of ulcer are often so intimate with the other forms of the disease that it becomes impossible to separate them. The condition known as "rodent ulcer," therefore, is a pronounced variety of milder epithelioma, and partakes of the very moderately malignant nature of this. And since there is no longer any doubt as to its identity, it will be proper for me at this time to put you upon your guard against falling into the error of imagining differences that do not exist. Remember, then, that the so-called "rodent ulcer" is simply an ulcer of the face, of chronic course and cancerous nature.

When the ulcer assumes the characters of infiltrating epithelioma, it reveals the action by involving, not only the skin and subcutaneous cellular tissue, but also the underlying muscles and bones. To these it becomes immovably attached. This tendency, indeed, may be present from the very first. Under such circumstances, the beginning may be, as in the flat form, from a wart or fissure, etc., or it may be from a small nodule imbedded in the skin. This slowly increases in size; new nodules arise about the margins, and there is formed a smooth, indurated, slightly elevated mass. In the meantime, the new growth is reaching down its prolongations into the corium, crowding out, in great degree, the blood-vessels, the sources of its own nutrition. Just as any tissue or individual, when deprived of its ailment, dies, so do these epidermic prolongations die when their vascular food-supplying tissues disappear before them. Thus, the dead epidermic masses, separate from those not yet sufficiently removed from the food-supply, and fall away from them. In this manner is formed the carcinomatous ulcer of whatever variety.

The infiltrating epitheliomatous ulcer now makes more rapid progress. It becomes deep, circular or oval, with hard unyielding edges, precipitous but not undermined. Its surface is granular or smooth, of a reddish or brownish varnished appearance, and discharges a small amount of ichor, which dries into a thin scab. The infiltration still stretches downwards, involving bones, muscles, whatever structures it meets, replaces them, and forms a solid unyielding mass. The boundaries of the ulcer continually enlarge, and frequently there appear nodules of waxy appearance, masses of epidermis cells—the advance guard of the main army, preparing to extend the widening surface of ulceration. The neighboring lymphatics no longer possess an immunity as in flat carcinoma; but infected with the now malignant process, become enlarged, hardened, and finally break down into ulceration, and become continuous with the original lesion, or enter upon a separate course of destruction.

Infiltrating epithelioma may destroy life in a few months When its course is less rapid, it may exist for some time, with its margin studded with nodules, or presenting a continuous border, with the waxy appearance of crumbly epidermic masses. This condition may last for a long time, but sooner or later, if not arrested by treatment, the progress of the disease will destroy life through exhaustion, mal-assimilation, pain, sleeplessness, and the accompanying train of disorders.

You will remember that I described the third case, that of Mrs G., as one of papillomatous epithelioma. There the tumor while not penetrating beyond the cutis, arose abruptly above the surrounding surface, not unlike a large button. Its slightly scarred surface was, except at a few small ulcerating points, covered with a smooth, thin epidermis. Papillomatous epithelioma may also assume the appearance presented by this very imperfect plaster cast. The patient was a woman forty-six years old, a laundress. The new growth had begun two years previously as a small pimple upon the dorsal surface of the metacarpo-phalangeal articulation of the left thumb. It gradually spread until it attained the dimensions seen upon the cast (five cm. transversely by three cm. antero-posteriorly). When I first saw it the appearance was that of a huge warty growth, as seen about the periphery; but as the margin had extended, a destructive ulceration of a very insignificant aspect had been going on in the center, visible only at scattered and minute points. The central cicatrix, the result of the healing of the ulceration, formed a pale, depressed surface of the size of a quarter of a dollar, surrounded by the centrifugal papillomatous elevations. This form of epithelioma may also appear in the course of the other two varieties, and may either be superficial or deep, or the three forms may co-exist. In any case it gradually and surely assumes the characters of the infiltrating form.

The *diagnosis* of epithelioma of the skin, usually need occasion but little difficulty. Syphilis, both in its initial and later manifestations, lupus, and finally simple warty growths, may be confounded with it. An infecting chancre may present characters not to be distinguished from cancer. The history, the rapidity of development, the acute course, and the early implication of the glands in the vicinity, usually enable us to distinguish a chancre; while the subsequent course of a cancerous ulcer, cannot allow us to remain long in doubt. A tertiary manifestation of syphilis may likewise cause hesitation; but here, again, there is the history, the usual concomitant conditions, the general appearance and course of the sore, whose evolution is rapid, inflammatory, actively suppurative, and readily amenable to appropriate internal medication. Lupus may be mistaken for epithelioma. But although lupus may be encountered in elderly persons, in them it will be found to have been present since youth, for lupus is essentially a malady of early life, though it may last indefinitely; while cancer of the skin, although occasionally seen in young people, is almost invariably a malady of advanced life, and rarely occurs before the fortieth year. The ulcer of lupus is superficial, without induration, and is usually accompanied by characteristic lupus tubercles. It, moreover, is not apt to invade osseous tissue. It is not always easy to decide between the scurfy accumulations, simple warts, etc., upon the faces and other parts of elderly people, and commencing cutaneous epithelioma. It must therefore, be a matter of the highest consequence to narrowly watch the course of such formations in the aged, especially where they first appear in advanced life; for who is able to recognize the transition stage, where benign merges into malignant new growth.

While it happens rarely that cutaneous epithelioma ends in spontaneous cicatrization and permanent recovery, it is by far the more usual experience to find it sooner or later assume all the features of malignancy and destroy life. We have seen how the superficial form often remains inactive for many years; on the other hand, life may be forfeited in a few months. But while skin cancer, if left alone, almost invariably pursues a fatal course, there can be no doubt that by appropriate interference it may be frequently arrested and permanently cured, especially in its earlier stages. It is true that one is often obliged to make more than one attempt to control the disease, and even then to encounter failure but too often. But by repeated destruction of the recurrent malady, one may fairly expect to finally triumph.

It follows that where the natural tendency of a disease is towards a fatal end, a large portion of the labor of medical and surgical minds should have been devoted to the task of devising relief from the dreaded scourge. There has resulted a multitudinous array of remedies—some worthless, others of more or less value. No disease has been attacked with a greater variety of remedies than cancer, both by internal and external application. I only desire to speak of preparations administered internally, as specifics against cancer, in order to condemn them. From time to time, the world has been startled by the discovery of a "new and certain cure for cancer," which, after a brief notoriety, has been consigned to the waste basket of experience.

Far otherwise is it with remedies locally applied; for at present there can be no doubt that epithelioma may be entirely and permanently cured by thorough removal of every particle of its structure from the tissues in which it is embedded. Unfortunately, this statement can only be made in a limited sense; for at best, it is usually necessary to practice more than one operation, and we can only hope to secure a permanent restitution to health in a portion of our cases. One reason for this is to be found frequently in the inaccessibility of the infiltrating masses. But apart from this, where this difficulty is not encountered, the healing art often fails, and the eye of the pathologist is unable to trace the path of the insidious invader.

The therapeutic agents at our disposal, are those which, by direct mechanical or chemical action, secure the removal of the infiltration. They have the same object in view, but attain it in different ways. With the scalpel, the mass may frequently be thoroughly removed. The same result may be attained with the dermal curette, which I now show you. Finally, the use of caustics will, nowhere more than in these cases, sometimes effect marvels.

Where the tumor or ulcer is situated upon an easily accessible part, where the loss of a large quantity of healthy integument with the resulting disfiguring cicatrix is of no moment, no one will question the greater value of the knife. The same instrument is, indeed, most appropriate in operations upon exposd parts, where the cancer is small, regular and well defined. But cutaneous epithelioma occurs, in the great majority of cases, upon that part of the body where deformity is most to be dreaded, and where small scars may produce the most unsightly disfigurement, namely, the face. Unfortunately, the knife cannot select the morbid from the healthy tissues, and in order to thoroughly remove every bit of the former, large portions of the latter must often be included. Thus is frequently incurred dreadful disfigurement without commensurate advantage. It is our duty, therefore, to search out agents capable of effecting the same results with the least possible destruction of sound structure.

The dermal curette or spoon, introduced into cutaneous therapeutics by Volkmann, of Halle, enables one to spoon out thoroughly all heterologous growth, while it is with the extremest difficulty and perseverance that one can tear out normal tissue. With it the crumbling elements of the cancer can be scraped away, without injuring the healty skin. It is a most valuable instrument, as I have found by experience, but I am inclined to think that its greatest usefulness is to be found in its employment as preliminary to other agents to be presently mentioned. It is, evidently, a difficult if not impossible task, to search out with the curette, all the elements of a cancerous infiltration tucked away in the interstices of healthy tissue; and, without doubt, one will frequently fail to effect their necessary complete removal.

Another agent of great value, is the actual cautery, employed either as the hot iron, electro-cautery, or, preferably, as the thermo-cautery of Paquelin. We have here, however, the additional disadvantage that the cautery has no choice between the tissues; and one can never know when the limits of the neoplasm have been exceeded. This objection also prevails against the use of the stronger potential caustics, such as potassa fusa, nitric acid, etc. These are, undoubtedly, unexceptionable agents for the destruction of tissue, but nothing can withstand their action. They involve all structures in a common ruin, and we are driven to employ various substances to check the destructive activity that we cannot otherwise control. Where, however, the object to be attacked is very small and circumscribed—in other words, where the epithelioma is in its earliest stages—these may be most profitably used.

It is, therefore, most fortunate that we possess other caustics whose more limited action renders them in these cases more valuable by far. As has already been remarked, with the knife and the most powerful caustics, it is often impossible to avoid far exceeding the limits of disease. This is especially the case where islets or tongues of healthy integument are enclosed by, or project into the new growth. The caustic agents I now wish to make you acquainted with, enable us, when we apply them, to destroy the morbid mass, without effecting any portion of healthy skin. By them, the cancer is dissected away with the greatest exactitude.

First of all, let me mention that mild caustic, which of late years has been almost banished from the class of escharotics—lunar caustic, silver nitrate. This agent may be appropriately fixed in a porte-caustique, or better still, in a goose quill, and its point driven into the morbid mass. It will, with gentle pressure, sink down until the healthy tissue is encountered. The point is now ploughed in all directions, wherever it will go—the epitheliomatous tissue melting away before it. When all of this has been destroyed, the point will be found to encounter unyielding resistance in every direction, and the operation is finished. If cicatrization be not complete at the end of a month, the process may be repeated. It will often be found advantageous to use silver nitrate after the dermal curette has removed all the grosser parts—a method I am about to put into practice upon the patient now before you.

It will frequently happen that a patient will not consent to submit to the necessary anæsthetization, or will refuse to submit to any operative procedure whatever. We still possess remedies that will effect our object in a slower manner. Arsenic may be used here in preference to other remedies. Mixed with an equal part of gum arabic, it may be applied, as Marsden's paste, and allowed to remain undisturbed for two hours or so. The resulting slough should be poulticed. I have, however, no experience with this procedure, and will not speak further of it.

I have had every reason to be gratified with the use of Cosme's arsenical paste as modified by Hebra, the composition of which I have already described. The method of its application, as recommended by that most distinguished dermatologist, and always observed by myself may be briefly described.

It having been decided to use this method (and in cases of moderate extent and intensity, it is most suitable), the surface of the epithelioma should be carefully cleansed. The paste, spread upon a piece of sheet lint to the thickness of a knife-blade, and corresponding to the surface of the new growth, should then be applied. A piece of oiled silk should be placed upon this, and over all a compress, held in position by adhesive strips. By the following day the patient will have begun to experience throbbing and some darting pain in the part, and upon removing the applications, the surface will be found to be swollen and reddened for some distance beyond the margins of the disease. After careful washing, the paste should be applied as before. Before the next dressing, which should be upon the following day, pain will have become very severe. By the end of the third day, or perhaps not until the fourth day, the parts will have become greatly swollen and reddened, and the surface of the epithelioma will be of a dark brown, charred appearance. The pain, which for the latter twenty-four or thirty-six hours will have been very severe, should be controlled by opiates. The redness and swelling need occasion no apprehension, since in a few hours after the removal of the caustic, they will disappear. A poultice should be applied until the slough begins to separate, which will be in a few days. At the expiration of a few weeks, this procedure may be repeated if any tendency to a recurrence of the disease be observed.

I have employed this treatment frequently, and have every reason to be satisfied with it. Its disadvantages are the protracted pain and the length of time required. The first we can control with anodynes; the second will often be preferred by the patient to the use of anæsthetics and the more speedy operation. The result is, in suitable cases, always equal, sometimes superior to that attained by the scalpel. The risk of arsenical poisoning is, here, practically *nil*.

Pyrogallic acid, in the form of a ten per cent. ointment, with vaseline or lard, has lately been recommended by Kaposi and others in the treatment of epithelioma, as a caustic agent; but, although I have used it, I am unable to give a decided opinion of its merits.

In conclusion, let me remind you that there are limits to our usefulness in this disease. Where there exists very great and wide-spread cancerous infiltration, or where the glands in the vicinity have become infected, we can rarely stay the progressive and destructive process. The enemy is a strenuous one, at best, and often defies our efforts. With the knife, the dermal curette, the actual and potential caustics, much may be accomplished in prolonging life, and in permanently overcoming the disease. But let it not be forgotten that the most good can be done when the disease is attacked early, vehemently, persistently.

A time will, nevertheless, often arrive, nay, may have already arrived when you are first consulted, when you may no longer hope to check the malignant advance. While powerless to cure the disease, or even to prolong life, in such cases, you may at least lend a supporting hand, and guide the failing footsteps along easy pathways, down into the dark valley.—*Virginia Medical Monthly.*

